# Language inclusion intentions in scoping reviews

**DOI:** 10.5195/jmla.2025.2170

**Published:** 2025-10-23

**Authors:** Joshua Wang, Hayley Moody

**Affiliations:** 1 reillyjj@qut.edu.au, Department of Research, Taipei Tzu Chi Hospital, Buddhist Tzu Chi Medical Foundation, New Taipei City, Taiwan; 2 h.moody@qut.edu.au, Learning and Teaching Unit, Queensland University of Technology, Queensland, Australia

**Keywords:** Language Bias, Evidence Synthesis, Scoping Review

## Abstract

**Objective:**

Research published in languages other than English (LOTE) is often ignored in evidence syntheses, marginalising diverse knowledge and global perspectives. While the extent of LOTE inclusion and the associated attitudes of LOTE inclusion amongst authors of systematic reviews has been well characterised, LOTE inclusion in other evidence synthesis forms has yet to be explored. Scoping reviews, in comparison to systematic reviews, examine a broader range of sources to build a conceptual summary of a field of inquiry, making LOTE literature an important source of information for scoping review authors. This study therefore aimed to characterise the current state of LOTE inclusion intentions in scoping reviews

**Methods:**

Peer-reviewed, PubMed indexed scoping review protocols published from 01-Jan-2024 to 11-Aug-2024 were analysed for LOTE inclusion. Author affiliation, which LOTEs (if any) were included, and what methods authors planned to use to read LOTE literature were recorded.

**Results:**

Overall, LOTE inclusion intentions and attitudes were diverse, with just under half of the 249 protocols analysed including a LOTE. Many LOTE-included articles relied on the authorship team's own LOTE proficiency to gather evidence. Machine translation was also intended to be used in one quarter of the LOTE-included protocols. Only 30% of the exclusive protocols planned to exclude LOTEs at the screening stage, allowing for readers to identify the number of LOTE articles.

**Conclusion:**

This analysis demonstrates the need for increased LOTE inclusion and reporting guidelines for scoping reviews, as well as the importance of analysing LOTE inclusion for other forms of evidence synthesis.

## INTRODUCTION

Evidence syntheses are widely considered the strongest level of evidence on which to base clinical practice and future research [1]. The reliability of evidence syntheses is built from their meticulous approach to include all relevant literature, including from the large body of research published in languages other than English (LOTE) [2–5]. However, LOTE evidence is often ignored in evidence syntheses [6–11]. The exclusion or neglect of LOTEs in systematic reviews is often due to a perceived lack of time, language expertise within the review team, or financial resources to utilise translation services [6,7].

The difficulty of LOTE inclusion has therefore prompted many scholars to ask: does the inclusion of LOTE literature in research syntheses searches warrant the additional effort required? This question has been indirectly addressed through two approaches. Firstly, comparisons of study quality between English and LOTE literature have been performed, with LOTE articles having either a lower [12,13] or similar [14–17] reporting completeness/study quality compared to English studies. Another group of studies has investigated whether LOTE exclusion influences the outcomes of meta-analyses. Studies found conflicting evidence that LOTE exclusion had no effect [18,19] or a significant effect [20] on the meta-analysis conclusions. Notably, LOTE exclusion consistently impacted meta-analysis findings in complementary/alternate medicine disciplines [17,21]. Overall, these approaches have failed to provide a definitive answer to the worthwhileness of including LOTEs in systematic reviews.

The above question may not be the most appropriate. Instead, the authors suggest approaching this topic with a lens of academic ethics and social justice: what effort should we as researchers make to include LOTE literature in our research syntheses? The development of English as an academic lingua franca is steeped in an Anglocentric past [22]. It continues to this day, where non-native English speakers must endure disadvantage throughout their academic careers [23]. Academics from the Global South are conditioned to believe that international/Western journals hold prestige unobtainable by local publications [24]. The databases used to assess publication quality exclude LOTE sources [25]. Google scholar relegates LOTE texts to the tail-end of its search results [26]. Meta-analysis conclusions remaining unchanged regardless of LOTE inclusion/exclusion does not demonstrate the futility of LOTE inclusion; it instead reflects the dominant anglophone research environment that non-native English speakers must endure. The authors therefore argue that LOTE inclusion is an ethical, rather than pragmatic imperative for evidence synthesis practitioners. Research continues to be published in LOTEs [27]; this research should be respected and considered.

While LOTE inclusion in systematic reviews has been analysed, the LOTE inclusion of other modes of evidence synthesis has been under-researched. Scoping reviews (ScRs) lay the foundations for other evidence syntheses by providing a broad overview of available research and grey literature related to a research area [28]. It is therefore arguable that ScR authors should be especially interested in LOTE literature which may provide unique approaches or insights that widen the review's findings. In comparison to the highly regulated nature of systematic reviews, ScRs have only recently been subject to some authoritative reporting standards and guidelines [29,30]. Therefore, there is little official guidance for language inclusion in ScRs outside of recommending that authors explain their reasons for any language restrictions [10,30]. To the best of the authors' knowledge, the only measure of ScR LOTE inclusion is from a 2020 editorial in *JBI Evidence Synthesis;* it states that in that particular journal issue, approximately half of the submitted ScR protocols did not exclude LOTEs [31]. Therefore, a more up-to-date and comprehensive analysis of LOTE inclusion in ScRs is needed.

This study aims to characterize the current intentions of ScR authors to include or exclude LOTEs in their reviews. ScRs are large undertakings and can take years to move from conception to publication. Therefore, analysing published ScRs would only provide dated insights into the final LOTE inclusion decisions made by authors. In contrast, examining recent ScR protocols provides a more current, accurate picture of how authors intend to approach language inclusion at the beginning of their studies [32]. Therefore, this study analyses ScR protocols as they are a contemporaneous source of language inclusion intentions. The results of this study can be used to help inform the development of ScR guidelines, promote language inclusion and ultimately diversify the perspectives in evidence syntheses. The specific objectives of this study are to:

1. Measure the proportion of ScR protocols that included at least one LOTE (LOTE-included) and don't include any LOTEs (LOTE-excluded)
2. Examine how language inclusion differs by the geographical affiliation of ScR protocol authors
3. Examine at what stage LOTEs are planned to be excluded in ScR protocols
4. Examine which LOTEs are most commonly included in ScR protocols
5. Determine how LOTE-included ScR protocol authors plan to translate LOTE literature
6. Determine if LOTE inclusion is associated with a multinational authorship team or the global/regional relevance of the ScR topic

## METHODS

### Data searching

ScR protocols cannot be registered in the International Prospective Register of Systematic Reviews, and therefore those that are made publicly available are either deposited to an online repository or published as an academic journal article [33]. For this study, we chose to only analyse ScR protocols published in peer-reviewed journals. Such articles not only represent the current language inclusion plans of ScR researchers, but they also likely represent the minimum language inclusion standards expected by the broader academic community (i.e. peer reviewers).

This paper aims to analyse the current state of language inclusion in peer-reviewed scoping review protocols. Therefore, the PubMed database was searched on the 11th August 2024 for results published in 2024 with the phrase “scoping review protocol” included in the title. Protocols in 2024 were analysed to provide an up-to-date summary of the language inclusion intentions of authors currently conducting scoping reviews. Inclusion of this title phrase as a search term is appropriate as it is an explicit component of scoping review protocol in authoritative guidelines [29]. Protocols published in journals that do not implement pre-publication peer-review, as indicated by the journals' instructions for authors, were excluded from the analysis. The search string used was: *(“2024/01/01”[Date - Publication]: “2024/08/11” [Date - Publication]) AND “scoping review protocol” [ti]*.

### Data extraction and analysis

A data extraction protocol was initially developed based on existing extraction forms for studies of language inclusion in systematic reviews [6,7]. A copy of the full data extraction is available (Appendix A). Restrictions on LOTEs can be employed at two distinct stages of the scoping review process: as part of the search strategy, or as part of the screening strategy. Excluding LOTE literature at the screening stage is preferred, as it gives a measurement of the number of LOTE articles that are excluded [34]. Language inclusion was classified within one of five levels:

1. *Language inclusion never mentioned*: the protocol gives no indication of the language/s included in the review (although the protocol itself is written entirely in English)
2. *LOTE excluded at the search stage*: the authors indicated that searches would be performed with a filter to return only English results.
3. *LOTE excluded at the screening stage*: the authors indicated that LOTEs would be excluded at the screening stage. This classification was also chosen if the protocol indicated LOTE exclusion generally and included a full example search string which did not filter the results to English only
4. *Unclear if LOTE excluded at the searching or screening stage*: LOTEs were excluded, but it was unclear at which stage
5. *LOTE included*: literature published in at least one LOTE was planned for inclusion in the scoping review if it was found during screening.

If the protocols did plan to include LOTE materials, then the method the authors planned to use in order to translate this material was also noted. If the protocols excluded LOTE materials, the presence/absence of an acknowledgement of the potential biases/limits this exclusion places on the ScR was recorded.

Multilingual search terms also influence the literature gathered for screening [35]. Therefore, data were also extracted on authors' intentions to use non-English search terms to locate literature. If this intention was present, the presence of multilingual search terms in example search strings was also noted.

Authorship teams with affiliations across multiple countries were more likely to include LOTE evidence in their systematic reviews [7]. Therefore, the countries of author affiliations were also recorded for each included protocol. It was also noted whether each protocol had an authorship team with affiliations from only one country, or from a multinational mix of countries. Hannah et al. [6] also proposes that reviews with a specific geographical scope could influence LOTE inclusion. We therefore examined whether each included protocol aimed to review literature with a specific regional focus.

All data extractions were performed manually using Microsoft Excel sheet templates. A random sample of 10 articles were used to pilot the initial data extraction protocol by both authors independently. The protocol was then refined to clarify any subjective aspects of the extraction protocol before data was extracted from all included articles. The completed data extraction sheets were then compared synchronously by both authors. Any discrepancies were resolved through discussion until a consensus was reached. Extracted data were analysed to produce descriptive statistics and graphical summaries. Chi-squared tests were performed using an online calculator [36] to examine the impact of geographical scope and international authorship on LOTE inclusion.

## RESULTS

### Overview of sample

**Figure 1:**
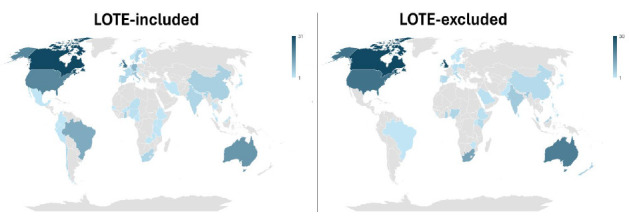
Geographic distribution of the author affiliations of LOTE-included (left) and LOTE-excluded (right) ScR protocols.

The search string without a publication year restriction returned 1696 PubMed indexed publications with “Scoping Review Protocol” in the title. A total of 262 (15.39%) of these records were published in 2024 and were further screened. One article was excluded as, while labelled a scoping review protocol, the full-text consisted of a fully completed scoping review. Two articles were excluded as they were corrections of previously published ScR protocols. One article was excluded as its full-text could not be located. Nine articles were excluded as they were published in journals which do not enact pre-publication peer review (Appendix B). Data was therefore extracted from the final 249 included articles (Appendix A). Generally speaking, the most prominent authorship affiliations were from the west, with Canada being the most common country of affiliation for authors (Figure 1; Table 1).

**Table 1:** Proportion of SCR protocols that are LOE-included for all countries affiliated with 10 or more protocols. For data on all countries, see Appendix C.

Country of Affiliation	Number of protocols	Number (%) of protocols that are LOE-included
Canada	61	31 (50.8%)
UK	50	22 (44.0%)
USA	41	19 (46.3%)
Australia	36	16 (44.4%)
South Africa	17	4 (23.6%)
Brazil	13	12 (92.3%)
Germany	12	10 (83.3%)
Ireland	11	6 (54.5%)
Denmark	10	8 (80.0%)
India	10	4 (40.0%)
Italy	10	7 (70.0%)

### Language inclusion intent

Of the ScR protocols analysed, 48.59% (n=121) included at least one LOTE (Table 2), while 51.41% (n=128) did not. Of the 128 LOTE-excluded protocols, 7 (2.81%) of the protocols did not mention language at all. Of those that explicitly stated that the scoping review would be limited to English only (n=121), 51 protocols (42.15%) planned to exclude LOTEs during their initial search. 38 protocols (31.40%) planned to include LOTEs in their search, and excluded LOTE literature during screening for inclusion/exclusion. 32 protocols (26.45%) stated that they would exclude LOTEs in their scoping review, but did not specify at what stage the exclusion would occur. No obvious differences between country of authorship affiliation were present between LOTE-included and LOTE-excluded ScR protocols (Figure 1). Of the 121 protocols that are LOTE-included, 13 (10.74%) stated that they would use LOTE search terms. Of these 13 protocols, 3 (23.08%) included LOTE search terms in the example search strategies in the ScR protocols.

**Table 2:** Count of language inclusion intentions

Intention	Count
LOTE included	121
excluded in initial search	51
excluded during screening search results	38
LOTE never addressed	7
unable to determine exclusion stage	32

### Languages included

Over half of the 121 LOTE-included ScR protocols (n=62, 51.24%) planned to include any/all languages in their review. For the other 59 protocols, specific LOTEs were planned for inclusion. In 43 (72.88%) of the protocols that specified one or more included LOTEs, the LOTEs included are commonly spoken languages in the author(s) affiliated country. Most of the protocols that named specific LOTEs (n=56, 94.91%) included 1-4 LOTEs, with one protocol each specifying 6, 7, and 11 LOTEs for inclusion (Table 3). The most common LOTEs included were French (n=24), Spanish (n=14) and German (n=13; Table 4). The other 128 ScR protocols did not include any LOTEs. Of these, 57 (44.53%) protocols discussed that the exclusion of LOTEs may limit and/or bias the findings of their review.

**Table 3:** Number of LOTEs included in LOTE-included ScR Protocols

number of LOTEs	Count
1	35
2	9
3	8
4	4
5	0
6	1
7	1
8	0
9	0
10	0
11	1

**Table 4:** Count of inclusion for each LOTE

Included LOTE	Count
Any language	62
French	24
Spanish	14
German	13
Portuguese	10
Swedish	9
Danish	8
Norwegian	8
Chinese/Mandarin	6
Italian	5
Other	20

### Translation strategies

Most of the LOTE-included ScR protocols mention how LOTE literature will be screened/read by the reviewer team (n=97, 80.17%). A majority of these ScR protocols (n=72, 74.23%) relied on the authors' LOTE proficiency to include a limited number of LOTEs into the ScR review. Machine translation was also reported as a common strategy (n=30, 30.93%), particularly for studies with an aim to include all LOTEs that appear during the ScR search. A comparatively smaller number of ScR protocols planned to use professional translation services (n=11, 11.34%) or collaborate with other researchers to translate/screen LOTE articles (n=6, 6.19%). A further 22 studies (22.68%) cited a combination of strategies, the most common of which was utilising author LOTE proficiency, followed by machine or professional translation for LOTEs which the review team has no proficiency (Table 5).

**Table 5:** Translation strategies for authors of LOTE-included ScR protocols

Translation strategy	Count
not mentioned	24
machine translation	30
collaboration with other researchers	6
hired translator	11
authors' ability	72
multiple strategies	22

### Influence of Geographical Scope and International Collaboration

It was hypothesised that LOTE inclusivity in ScR protocols may be influenced by the geographical scope of the review, or the presence of an international authorship team. Of the 165 protocols affiliated with a single country, 81 (49.09%) were LOTE-included. Of the 84 protocols with an international authorship team, 36 (42.86%) were LOTE-included. No significant relationship was present between the LOTE inclusivity of ScR protocols, and whether the authors had affiliations from multiple countries (X2=0.8684, df=1, p-value=0.351). Of the 66 protocols with a specified geographic scope, 31 (46.97%) were LOTE inclusive. Of the 183 protocols without a specified geographic scope, 90 (49.18%) were LOTE-included. The presence/absence of geographical scope in the ScR protocols has no significant relationship with LOTE inclusivity (X2=0.0949, df=1, p-value=0.758).

## DISCUSSION

This study represents the first broad examination of LOTE inclusion in scoping reviews. Overall, just under half of peer-reviewed ScR protocols published in 2024 and indexed in PubMed planned to include at least one LOTE. This frequency of LOTE inclusion is similar to those reported in analyses of published systematic reviews [6,8,11]. This similarity is surprising given that ScRs, unlike systematic reviews, are aimed at providing a broad overview of a field, often by incorporating grey literature. Furthermore, we anticipated that the rate of planned language inclusion would be higher in ScR protocols compared to published evidence syntheses, given that more language-inclusive strategies may be abandoned during the implementation phase of a review. However, given that over half of the ScR protocols analysed plan to exclude all LOTEs, this study demonstrates an enduring divide in attitudes towards language inclusion in evidence syntheses.

A divide also exists in the depth of reporting LOTE exclusion. Firstly, 32 of the 128 LOTE-excluded protocols planned to exclude LOTEs at the screening stage. This approach allows readers to identify the number of LOTE articles to be excluded, or to enable future translations that may provide additional information to the findings. The remaining 96 protocols (75%) either did not specify when they would exclude LOTEs, or excluded LOTEs as an in-built search filter. Therefore, a majority of evidence synthesis authors are currently not aligning with best-practice language exclusion reporting [34]. Excluding LOTEs at the screening stage allows readers to evaluate the amount of excluded LOTE literature. Transparently reporting the excluded LOTE literature would also allow other researchers to perform additional analyses on this literature. Therefore, scoping review guidelines should more clearly recommend LOTE exclusion at the screening stage to improve the prevalence of best practice. Additionally, less than half of the LOTE-excluded protocols acknowledged the limitations of their approach in the protocol.

Contradictory to Rasmussen and Montgomery [7], our findings suggest that multinational authorship teams are not, necessarily, more likely to be language inclusive in their ScRs. This result may be due to the large representation of authors from bilingual nations in the sample (e.g. Canada). Hannah et al. [6] found that ecological systematic reviews with a specific geographic scope were associated with greater LOTE inclusion. Our results instead found no significant difference in LOE inclusion when comparing scoping review protocols on global or local issues. The discrepancy between these studies may be due to disciplinary differences, or differences in the determinants of LOE inclusion between systematic and scoping reviews.

Approximately half of the LOTE-included protocols analysed in this study aimed to include any/all LOTEs in their ScR, usually through relying on translation methods external to the authorship team such as machine translation or hiring professional translators. Despite general hesitation towards adopting machine translation and language exchange systems reported by Hannah et al. [6] our findings do demonstrate a willingness to utilise machine translation methods, and collaborative approaches with peers external to the research team in order to include LOTE literature. The high proportion of protocols planning to use machine translation is important to note given that, unlike the highly defined data extraction of systematic reviews, the potential diversity of ScR data extractions may require more nuanced language ability [37]. While machine translation provides a free and accessible alternative to professional translation services, researchers may struggle to correctly interpret translations produced through this software. Future analyses of published LOTE-included scoping reviews that have interpreted LOTE literature using machine translation could determine if they are a viable tool for LOTE-included scoping reviews.

Overall, our analysis demonstrates that the prevalence of language inclusion in ScRs can be improved. Our results suggest that the majority of ScRs only consider English-language sources, and those that plan to examine LOTEs are usually restricted to LOTEs commonly spoken in the author(s)' country. More detailed guidelines on language inclusion in ScRs (beyond justifying language restrictions) may assist researchers in expanding the languages considered in their evidence syntheses. We recommend that ScR guidelines also explicitly state that LOTE-excluded protocols should exclude LOTE literature at the screening stage. More broadly, our analysis reveals that efforts to support LOTE scholarship and scholars should continue and be directed towards other forms of evidence synthesis aside from systematic reviews.

## FUTURE DIRECTIONS

Language inclusion in ScRs and ScR protocols are under researched when compared to other forms of evidence synthesis like systematic reviews. Given that ScRs rely on gathering wide-ranging approaches and findings to inform the design of future research, it is possible that LOTE exclusion in ScRs is more likely to compromise the review findings when compared to LOTE exclusion in systematic reviews. Therefore, in a fashion similar to Nussbaumer-Streit et al. [19] LOTE-excluded ScRs could be re-analysed in the future to include LOTE literature, followed by an analysis of if/how this retroactive LOTE inclusion changes the outcomes of the ScR. Additionally, ScR protocol authors from the existing dataset developed here could be surveyed to better understand their LOTE inclusion intentions and barriers/concerns towards LOTE inclusion [7]. While our study has examined the lack of association between LOTE inclusion and international collaboration, we rarely encountered explicit explanations for the LOTE inclusion/exclusion. Directly surveying or interviewing ScR authors would therefore address why certain LOTE inclusion decisions are made.

Additional unobtrusive data could also be gathered to better understand authors' language inclusion intentions. This could include extracting data on the planned use of predominately non-English databases (e.g. CNKI) in the protocol's proposed search strategy. Additionally, language inclusion could potentially be mandated by research funding sources. Future research in this field could therefore examine if LOTE inclusion is correlated with any particular policies of the research funder/s. More granular analyses of the appropriateness of the LOTE inclusion strategy for addressing the research questions of each of the ScRs could also be performed. Lastly, how ScR authors ultimately enact or abandon the intentions they record in ScR protocols is unknown. Future research should therefore examine how LOTE inclusion shifts between ScR protocols and the completed ScRs that result from them.

## LIMITATIONS

The search strategy utilised may have limited the final dataset gathered. Although it is recommended in authoritative guidelines to label ScR protocols as “Scoping Review Protocols” in the manuscript title [33], some ScR protocols may not have done this and would therefore have been missed during the search. Additionally, only the PubMed database was searched; it is possible that some biomedical journals publishing ScR protocols are not PubMed indexed and therefore would have been missed in the initial search. An expanded search of multiple databases, especially those based outside of the US or those that include predominately non-English texts, would strengthen the generalisability of our findings. Lastly, while peer-reviewed ScR protocols were chosen since they also reflect peer-reviewer standards/attitudes towards LOTE inclusion, excluding non-peer-reviewed ScR protocols deposited in online repositories likely biases the analysis. Most of the ScR protocols analysed were published in journals that charge article processing charges in excess of $1,000 USD for a scoping review protocol. Given that many marginalised scholars cannot afford this expense [38,39] our dataset is likely skewed towards scholars from more economically privileged environments.

## Data Availability

The raw data associated with this article are available in Appendix A.
