# Supplementary material for: Language inclusion intentions in scoping reviews
Source: J Med Libr Assoc. 2025 Oct 23;113(4):290–7. doi: 10.5195/jmla.2025.2170 (PMC12604074; doi:10.5195/jmla.2025.2170)
Supplement: Supplementary file 2 — Appendix B [file jmla-113-4-290-s02.docx]

Appendix B: Overview of Journals publishing ScR Protocols

| Included | |  | Excluded – post-publication peer review venues | |
| --- | --- | --- | --- | --- |
| Journals with included protocols | Number of included protocol papers |  | Excluded journal name | Number of excluded papers |
| BMJ Open | 112 |  | HRB Open Res | 4 |
| JBI evidence synthesis | 53 |  | Research Square | 2 |
| PLoS One | 45 |  | Open Res Eur | 2 |
| Systematic Reviews | 11 |  | F1000Res | 1 |
| Other*: | 28 |  |  |  |
| Total: | 249 |  | Total: | 9 |
| *Consists of 2 protocols published in Acta Anaesthesiology Scand, 2 protocols published in Nursing Reports, and 1 protocol published in each of the following journals: Anaesthesiology Research Practice, BJGP Open, BMJ Paediatric Open, British Journal of Nursing, Campbell Systematic Reviews, Canadian Medical Education Journal, Clinical Exp Allergy, Digital Health, Disability Rehabilitation Assist Technol, Frontiers in Public Health, Health Science Reports, International Journal of Circumpolar Health, International Journal of Environment Research Public Health, International Journal of Lang Commun Discord, International Journal of Nursing Education Scholarship, International Journal of Surgical Protoc, Journal of Evaluation in Clinical Practice, Journal of Family Medicine and Primary Care, Medwave, Methods Protoc, Regen med, Res Involv Engagem, Resusc Plus, South African Journal of Infectious Disease. | | | | |
