# Supplementary material for: Language inclusion intentions in scoping reviews
Source: J Med Libr Assoc. 2025 Oct 23;113(4):290–7. doi: 10.5195/jmla.2025.2170 (PMC12604074; doi:10.5195/jmla.2025.2170)
Supplement: Supplementary file 3 — Appendix C [file jmla-113-4-290-s03.docx]

Appendix C: Proportion of ScR protocols that are LOE-included by author-affiliation.

| Country of Affiliation | Number of protocols | Number (%) of protocols that are LOE- included |
| --- | --- | --- |
| Canada | 61 | 31 (50.8%) |
| UK | 50 | 22 (44.0%) |
| USA | 41 | 19 (46.3%) |
| Australia | 36 | 16 (44.4%) |
| South Africa | 17 | 4 (23.5%) |
| Brazil | 13 | 12 (92.3%) |
| Germany | 12 | 10 (83.3%) |
| Ireland | 11 | 6 (54.5%) |
| Denmark | 10 | 8 (80.0%) |
| India | 10 | 4 (40.0%) |
| Italy | 10 | 7 (70.0%) |
| Ghana | 9 | 5 (55.6%) |
| China | 8 | 5 (62.5%) |
| Netherlands | 8 | 5 (62.5%) |
| Korea | 7 | 3 (42.9%) |
| Malaysia | 7 | 2 (28.6%) |
| Spain | 7 | 4 (57.1%) |
| Nigeria | 6 | 2 (33.3%) |
| Portugal | 6 | 3 (50.0%) |
| Switzerland | 6 | 4 (66.7%) |
| New Zealand | 5 | 0 (0.0%) |
| Sweden | 5 | 4 (80.0%) |
| Kenya | 4 | 1 (25.0%) |
| Chile | 3 | 3 (100.0%) |
| Ethiopia | 3 | 1 (33.3%) |
| France | 3 | 3 (100.0%) |
| Japan | 3 | 2 (66.7%) |
| Luxembourg | 3 | 1 (33.3%) |
| England | 2 | 0 (0.0%) |
| Hong Kong | 2 | 0 (0.0%) |
| Niger | 2 | 2 (100.0%) |
| Norway | 2 | 1 (50.0%) |
| Philippines | 2 | 0 (0.0%) |
| Poland | 2 | 0 (0.0%) |
| Qatar | 2 | 0 (0.0%) |
| Saudi Arabia | 2 | 0 (0.0%) |
| Taiwan | 2 | 0 (0.0%) |
| Uganda | 2 | 1 (50.0%) |
| Austria | 1 | 1 (100.0%) |
| Belgium | 1 | 1 (100.0%) |
| Burkina Faso | 1 | 1 (100.0%) |
| Cameroon | 1 | 1 (100.0%) |
| Colombia | 1 | 1 (100.0%) |
| Cyprus | 1 | 0 (0.0%) |
| Czech Republic | 1 | 1 (100.0%) |
| Ecuador | 1 | 1 (100.0%) |
| Finland | 1 | 1 (100.0%) |
| Gambia | 1 | 1 (100.0%) |
| Iran | 1 | 1 (100.0%) |
| Lithuania | 1 | 1 (100.0%) |
| Malta | 1 | 1 (100.0%) |
| Mexico | 1 | 1 (100.0%) |
| Nepal | 1 | 1 (100.0%) |
| Pakistan | 1 | 0 (0.0%) |
| Peru | 1 | 1 (100.0%) |
| Saint Kitts and Nevis | 1 | 0 (0.0%) |
| Scotland | 1 | 0 (0.0%) |
| Senegal | 1 | 1 (100.0%) |
| Servia | 1 | 1 (100.0%) |
| Sierra Leone | 1 | 0 (0.0%) |
| Sri Lanka | 1 | 0 (0.0%) |
| Tanzania | 1 | 1 (100.0%) |
| Thailand | 1 | 1 (100.0%) |
| Zambia | 1 | 1 (100.0%) |
